# Temperature and Humidity Stability of Fibre Optic Sensor Cables for High Resolution Measurements

**DOI:** 10.3390/s23031296

**Published:** 2023-01-23

**Authors:** Marcus Maier, Cedric Kechavarzi, Xiaomin Xu, Janet M. Lees

**Affiliations:** 1 Department of Engineering, University of Cambridge, Cambridge CB3 0FA, UK; 2 Centre for Smart Infrastructure and Construction, University of Cambridge, Cambridge CB3 0FA, UK

**Keywords:** distributed fibre optical sensors, temperature, strain, calibration, concrete, humidity

## Abstract

Fibre optic sensors offer a means for the real-time continuous measurement of temperature or strain in concrete structures. Backscattered light along a fibre optic sensing (FOS) cable is interrogated to record a frequency shift and this shift is typically translated into a physical parameter such as strain or temperature using a calibration factor. However, when the measured frequency shift is a response to a combination of mechanical, thermal or hygral (humidity) loadings it is difficult to decouple individual influences. This presents a challenge in complex materials such as concrete where the strain, temperature and moisture levels change concurrently during the fresh and hardened states. Furthermore, depending on the application, both short- and longer-term measurements are required. As such, not only is the influence of these physical factors of interest but also the time and spatial stability of the measured frequency, which is highly dependent on the FOS cable composition. To investigate this aspect, fibre optic cables commonly used for strain (three tight-buffered cables) or temperature (two loose-buffered cables) measurement were considered. The cables were subjected to mechanical or environmental exposure and interrogated using a high-resolution optical backscatter reflectometer. The exposure regimes included three temperature cycles with sustained steps from 10 °C to 60 °C and back to 10 °C and an increasing and decreasing humidity cycle with steps between 30 to 90% relH. These ranges were selected to be indicative of typical environments for concrete. The results showed that the calibration factors back-calculated from increasing and decreasing temperature or humidity cycles differed. The third temperature cycle results were found to exhibit the smallest differences between heating and cooling suggesting that temperature pre-conditioning prior to installation could be advantageous. For all the cables, a drift in the readings was observed over the duration (2.5 h for temperature and 30 h for moisture) of the sustained steps. The magnitude of the drift depended on the cable type and exposure condition. In addition, local frequency fluctuations along the cable were observed which would need to be taken into account if only a single point along the cable length was used for analysis. The obtained results highlight the importance of the cable selection to maximise the FOS measurement fidelity for a given parameter of interest.

## 1. Introduction

Structural health monitoring (SHM) using distributed optical fibre sensors (DFOS) is gaining popularity in civil and marine engineering applications. Fibre optic sensors have been used to monitor parameters such as strain and temperature in large buildings, piles, bridges, tunnels and offshore platforms [1,2,3,4,5,6] where FOS cables are mounted or embedded into the structure. Accurate FOS measurements are key in order to evaluate and predict the physical behaviour. However, in a complex material such as concrete the FOS measurements are influenced by variations in the intrinsic and ambient temperature, moisture and strain conditions. Knowledge of intrinsic changes are of importance in understanding phenomena such as the hydration of concrete, autogenous and drying shrinkage and concrete strength development. Harsh environmental exposure e.g., in marine and offshore structures due to wave impact and changing sea levels, also induces a combination of strain, temperature and moisture conditions in concrete structures. The influence of intrinsic and environmental exposure on FOS measurements must therefore be evaluated and analysed to evaluate the measurement accuracy and draw comparisons with traditional sensors. These interactions also depend on the cable type. Thus, an awareness of the influence of temperature, strain and moisture on FOS measurements derived for a given cable type is necessary to inform the selection of FOS cables that are most likely to provide high quality data and an accurate measurement of parameters of interest.

The principle behind DFOS is that some properties of the components of the backscattered light spectrum during forward propagation of an optical pulse in the core of optical fibres can be correlated to strain or temperature changes. The advantage of DFOS lies in the ability to measure continuously along the fibre length instead of at discrete pre-defined points, as would be the case with conventional strain gauges or thermocouples. An additional benefit is that fibre optic cables are immune to electro-magnetic interference [7,8].

The backscattered light profiles are typically analysed using Brillouin, Raman or Rayleigh scattering. These analysis methods differ in terms of spatial resolution, accuracy and the length over which the signal can be measured. To date, Brillouin scattering has been the most studied technique as measurements over lengths of several kilometres with a single cable are possible. This suits the SHM of large structures where a high spatial resolution is not necessarily required. Strains and temperatures can be recorded within increments of tens of centimetres to metres. But analysis approaches such as the pre-pulse pump (PPP) Brillouin technique can enable spatial resolutions as low as 2 cm [9]. Raman backscattering has a spatial resolution of 1 cm to several metres for cable lengths of up to 40 km [8]. However, it is a thermally activated process so the Raman analysis only allows for temperature but not strain measurement which limits wider applications in civil engineering [10,11]. Recent improvements in Rayleigh backscattering instruments have led to submillimetre spatial resolutions and measurement lengths of several metres (up to 50 m). Hence Rayleigh techniques are of particular interest for monitoring strains and temperatures in mid- and small-scale concrete elements [7].

The selection of the FOS cable also influences the measurement results. Based on the parameter under investigation different cables tend to be preferentially suited for either strain or temperature measurement. For strain measurement so called tight-buffered cables are common, where the optical fibre (core, cladding and coating also referred to as the ‘bare’ fibre) are tightly connected to further buffer and jacket protective coating layers to promote strain transfer to the fibre core. The cable composition influences the sensitivity, distribution and magnitude of the measured strains. For instance, the coating thickness and differential elastic and inelastic deformations between coating layers during mechanical loading affects the sensing properties of the cable [12]. A stiffer coating transfers higher strains to the fibre core [13]. Slip between the coating layers can result in inaccurate strain measurements [14]. For temperature measurement so called loose tube cables are typically used. In such cables the fibre core is guided within a tube filled with gel or a hollow tube with an air cavity to decouple the core from the outer protective layers. This helps to ensure that the fibre core is primarily affected by temperature changes. Studies on temperature measurements with FOS are mostly validated against conventional thermocouple readings [15,16]. For instance, Ge et al. [15] embedded a loose tube FOS in a reinforced concrete beam subjected to thermal loading of up to 45 °C and noted a difference of 2–5 °C between the FOS and thermocouple readings.

In concrete applications, material insights are often best characterised using a tight spatial resolution and a stable and stringent measurement accuracy. The high spatial resolution offered by the Rayleigh technique has therefore been exploited using DFOSs that are either embedded within concrete during casting, or adhered to a hardened concrete surface [17,18,19,20,21,22,23,24]. However, strain, temperature and humidity exposure can all lead to frequency changes in the DFOS signal, and reliably decoupling these individual influences is difficult. In addition, the cable composition itself influences the strain and temperature measurement, but there is currently limited or no calibration data to establish the influence of multiple simultaneously changing environmental conditions on the calibration and measurement accuracy. A further complication with DFOS are temperature and strain reading anomalies (TRA [25] and SRA [24,26]) where unrealistic magnitudes of strains or temperatures are observed at random locations along the cable length. This is potentially a further hindrance in concrete applications where moisture, temperature and strain conditions can all vary in a time dependent manner. These effects, individually or collectively, can lead to frequency shifts in the DFOS response that are exacerbated by non-linearities in the fibre optic cable response to temperature, moisture and strain. A particular challenge is therefore how to accurately assess these effects within data derived from FOSs analysed using high resolution interrogators.

This paper investigates FOS cables subjected to step changes in either strain, temperature or humidity sustained under controlled conditions. The frequency response was measured using an optical backscatter reflectometer (OBR) and the protocols reflected mechanical or environmental ranges of exposure that could be expected in concrete applications during the fresh or hardened state. The in-depth calibration tests were conducted on tight buffered and loose tube cables and the time-dependent and longitudinal stability was studied to identify local frequency deviation. The results were also compared with the temperature and humidity readings with the calibration chamber to ascertain measurement inaccuracies and their influence in the FOS measurement of temperature or moisture. The obtained results will help in the selection of appropriate cables for strain, moisture and temperature measurement within concrete, in situations where these conditions change significantly over time (e.g., during hydration or in marine and offshore structures). Furthermore, the results will raise awareness of the differences between FOS derived temperature and strain measurement results compared to those acquired using traditional sensors such as thermocouples and strain gages.

## 2. Experimental Programme and Measurement

A total of six fibre optic cables (a bare fibre, 3 tight-buffered strain and 2 loose-tube temperature cables) were investigated and subjected to mechanical or environmental exposure. The applied strain (0 to 2000 με), temperature (10 to 60 °C) and humidity (30 to 90% rel. H) ranges were selected to be indicative of typical environments for concrete.

### 2.1. FOS Cables

**Figure 1:**
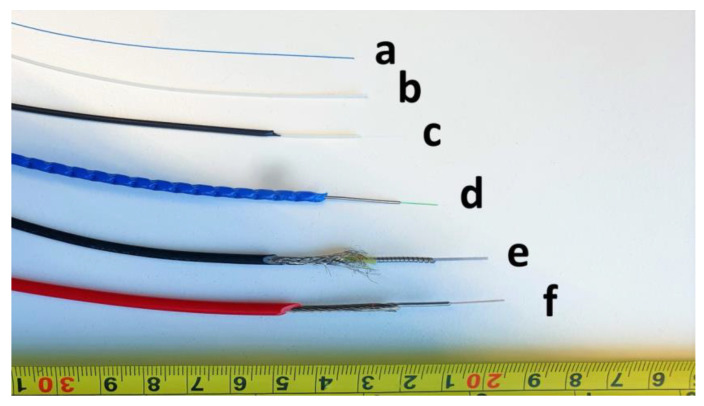
Cable types (a) bare fibre, (b) 0.9 mm tight buffered, (c) 2.0 mm tight buffered, (d) 3.2 mm ribbed tight buffered, (e) 3.0 mm air filled loose tube cable (f) 3.8 mm gel filled loose tube cable.

Based on the Manufacturer’s data sheets for each cable, the fibre optic diameter, cable construction, coating, weight and minimum bend radius are presented in Table 1 and a photo of the cables is shown in Figure 1. A 125 μm single mode fibre with a 250 μm acrylate coating was used as a reference sensor (bare fibre). The tight buffered ‘strain’ cables (denoted as ‘TBC’ cables) included a smooth 0.9 mm outer diameter cable with a single fibre locked to a Hytrel (TPC-ET thermoplastic elastomer) outer sheath, a smooth 2.0 mm cable consisting of the 0.9 mm cable above with an additional layer of polyurethane (PU) outer sheath, and a ribbed 3.2 mm outer diameter cable with a single fibre bonded inside a central metal tube embedded in a structured polyamide (PA) outer sheath. The loose tube ‘temperature’ cables (referred to as ‘LBC’ cables) were a 3.8 mm outer diameter cable consisting of two fibres in a central gel filled stainless-steel loose tube protected by a stainless-steel wire armour and an outer PA sheath, and a 3.0 mm outer diameter cable made of a single fibre in an air-filled plastic loose tube inside a coiled stainless-conduit protected by a woven steel fabric and a low-smoke-zero-halogen (LSZH) thermoplastic polymer outer sheath. The cable lengths that were available for the study are also shown in Table 1.

### 2.2. Calibration Test Specification

**Figure 2:**
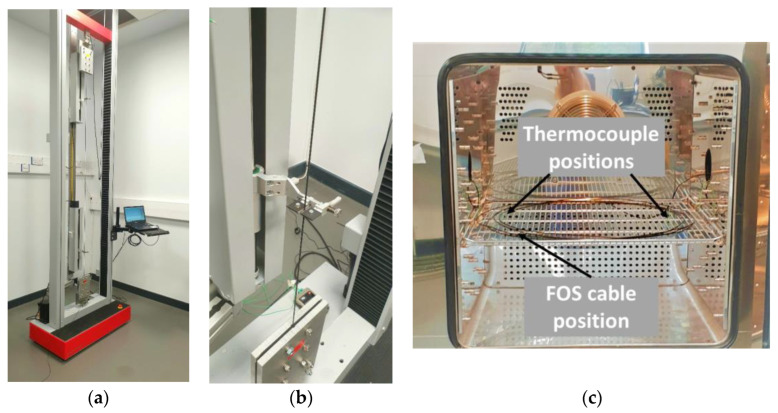
Photo of (**a**) overview of tensile test set-up and (**b**) plate grip and extensometer clips and (**c**) environmental chamber with indicated positions of the thermocouples.

**Figure 3:**
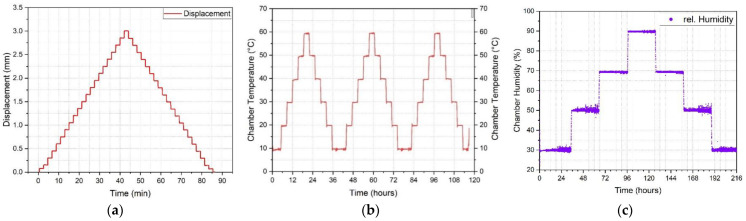
(**a**) displacement cycle 0–3–0 mm (0–2000 με) for strain calibration tests (**b**) heating and cooling cycles (10–60–10 °C) for temperature calibration tests and (**c**) relative humidity cycle (30–90–30%) for humidity calibration tests.

The strain calibration was performed using a universal testing machine (Testometric Ltd., Rochdal, UK) with a frame height of 3.7 m and a maximum gauge length of 2.5 m and a load capacity of 10 kN. Plate grips were designed to hold the cables in place without slippage during testing. The two steel plates (height by width = 220 × 200 mm^2^) had grooves covered with a rubber sheet in which the cable was guided to avoid slippage while only gripping the outer sheath and preventing crushing of the cable. Two clip-on extensometers, placed at a distance of 1500 mm apart (gauge length) on the tested cable (with a total length of at least 4.5 m), measured the displacement during testing. A photo of the experimental set-up can be found in Figure 2a,b. The tensile calibration tests were performed with a displacement cycle of 0–3–0 mm with 20 steps of 0.15 mm (see Figure 3a). This equates to a maximum strain of 2000 με with 20 steps of 100 με. Each displacement level was held for 2 min before the next level was attained with a loading speed of 0.1 mm/s. The total duration of the strain calibration test was 80 min.

The same set of cables were used for the temperature and humidity calibration tests and were laid loosely in coils inside a Memmert CTC-256 environmental chamber (see Figure 2c). The chamber allows for the simultaneous control of temperature between −42 °C and +190 °C and relative humidity between 10% and 98%. The chamber also records the internal temperature and humidity. The fan speed was set to 70% to help ensure a constant temperature and humidity distribution throughout the climatic chamber. For each cable type, the entire cable was placed in the chamber but with an offset length of between 1 m and 2.1 m from the analyser (see Table 1).

For the temperature calibration a heating and cooling regime of 10–60–10 °C (see Figure 3b) with 10 °C steps at a constant relative humidity of 50% was used. Each temperature step was held for 3 h. A heating rate of 0.5 °C/min for each temperature step resulted in a total testing time of 36 h per temperature cycle and a total testing time of 108 h (~4.5 days). Three cycles were performed for each fibre optic cable. Two thermocouples were placed on the left and right side inside the chamber, close to the fibre optic cable, to provide an independent measure of temperature and to check the temperature distribution at different locations within the chamber (see Figure 2c).

For the humidity calibration, a relative humidity range of 30–90–30% was applied with 20% steps at a constant temperature of 30 °C (see Figure 3c). Each relative humidity step (30–50–70–90–70–50–30%) was held constant for 30 h, the increase/decrease to the next humidity level was completed within 30 min (0.67% rel. H/min). This resulted in a total calibration time of 210 h (~8.8 days) for one humidity cycle.

An example of the recorded displacements from the universal testing machine, thermocouple temperatures within the chamber and chamber humidity profiles are shown in Figure 3. The readings suggest the behaviour is broadly in line with the desired calibration regimes. The deviations in the chamber temperature were within ±0.2 °C although humidity fluctuations of up to 6.0% were evident at 30% and 50% humidity levels.

### 2.3. FOS Data Capture and Analysis

The FOS data were recorded with an optical backscatter reflectometer (OBR) from Luna Technologies (Model ODiSi-6104). The interrogator defines virtual gages along the sensor cable in which it averages and analyses the backscattered signal. The length of the virtual gage (called ‘gauge pitch’) can be individually set and was chosen to be 2.6 mm for all the presented test results with a sampling rate of 1 min for the temperature and humidity calibration test and 1 s for the strain calibration tests.

## 3. Evaluation of the Cable Calibration Factors

The FOS cable calibration factor links the recorded frequency shift which occurs during mechanical, thermal or hygral loading to either strain, temperature or humidity. It is obtained by plotting the frequency shift over the strain, temperature or humidity changes and performing a linear regression through those datapoints. The slope of the regression line represents the calibration factor. In the following readings at selected times and points along the length of the cable are taken as indicative of an ‘average’ response and used to determine best fit calibration factors for a given cycle. The influence of time-dependent frequency fluctuations under constant exposure conditions and frequency variations along the cable length are then investigated.

### 3.1. Calibration Factor for Strain Exposure

To find the average strain calibration factor for the increasing and the decreasing strain cycle, the selected datapoints described in Table 2 were used as the basis for a linear regression analyses. With the exception of the bare fibre (due to cable length restrictions), the data points sampled for analysis were spaced at 0.25 m along the cable length located between the two extensometer clips. The data were extracted every 10 s during the 80 min of testing. The resulting total number of datapoints for each increasing and decreasing strain cycle is given in Table 2 together with the cable length, spatial increment along the cable and the sampling rate.

**Figure 4:**
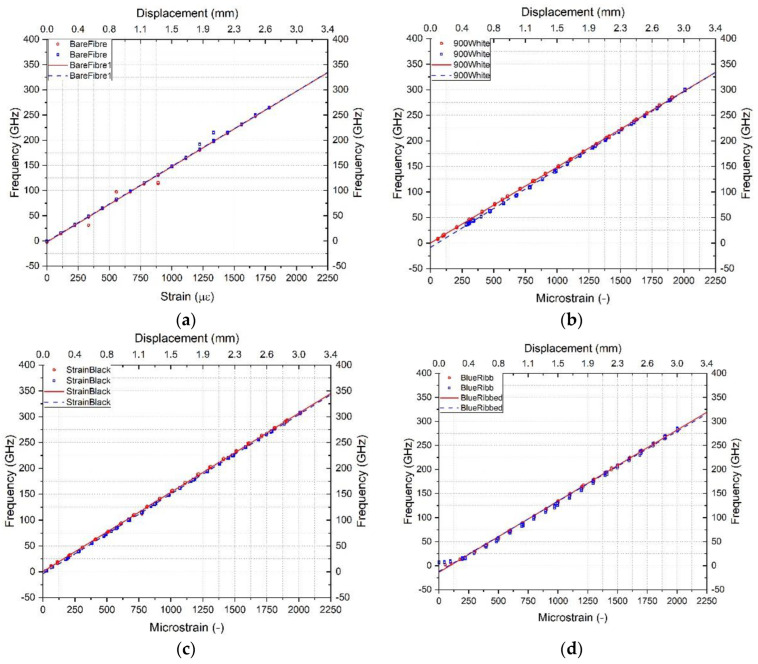

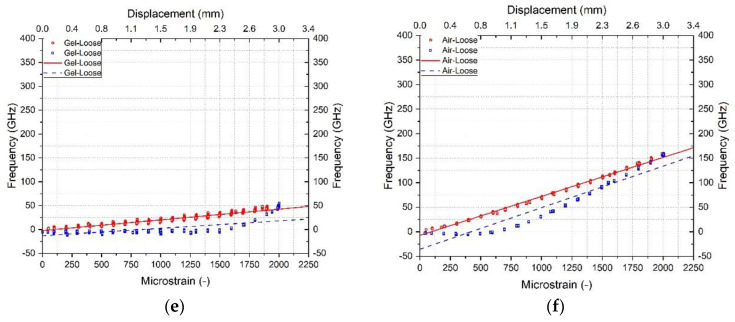
Increasing and decreasing strain calibration datapoints and linear fit of (**a**) bare fibre, (**b**) 900 μm tight buffered, (**c**) 2.0 mm tight buffered, (**d**) 3.2 mm ribbed tight buffered, (**e**) 3.8 mm gel filled loose tube and (**f**) 3.0 mm air filled loose tube cable.

Figure 4 shows the increasing strain level datapoints in red, the decreasing strain levels in blue and the linear regression best fit lines. The bare and tight buffered cables (Figure a–d) show good linearity and low hysteresis between the increasing and decreasing strain cycles. On this basis they appear to be suitable for strain measurement. In the loose tube cables, the optical fibre is decoupled from the other cable components to minimise the influence of mechanical strain on the cable jacket i.e., to decouple the temperature measurement. It can be seen in Figure 4e,f that the loose tube cables exhibit a frequency increase even for small increasing strain levels. During unloading (from 2000 to 0 με) the frequency decreases initially and reaches a plateau at below ~1500 με or ~600 με for the gel filled and air filled loose tube cables, respectively. This suggests that the fibre core does not pick up strain below this strain level but does experiences strain above that level. The strain calibration factors for loading or unloading obtained from the linear regression analysis are summarised in Table 3 along with the average calibration factor that encompasses the combined loading and unloading stages. The difference between the increasing and decreasing strain cycles is lowest for the bare fibre and the 2.0 mm tight buffered cable (TBC) with a difference of 0.7%, followed by the 3.2 mm ribbed TBC with 1.4% and highest for the 0.9 mm TBC with a 2.7% difference. Due to the different behaviour between the increasing and decreasing loading cycles the loose tube cables (LTC) showed differences of 7.6% (3.0 mm air filled LTC) and 27.3% (3.8 mm gel filled LTC).

### 3.2. Calibration Factor for Temperature Exposure

**Figure 5:**
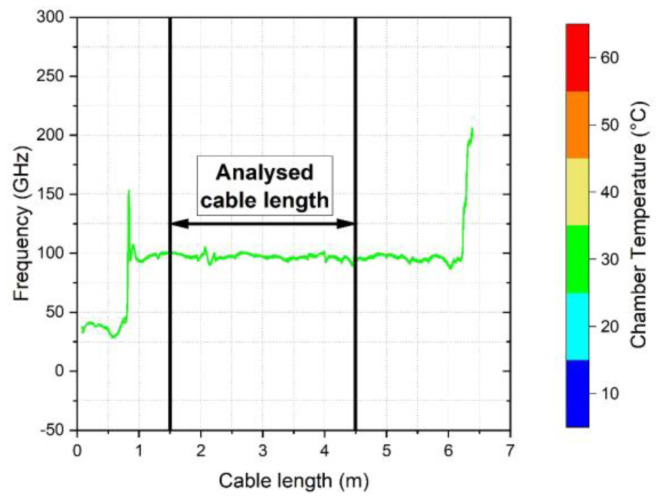
Representation of the selected 3.0 m cable length used to analyse the temperature calibration factor. Peaks at the 1.0 m and 6.2 m show the position where the cable is fed into the chamber and the end of the cable, respectively.

Data from a 3.0 m long cable section positioned within the heating chamber (see Figure 5 for an example) was analysed to assess the temperature calibration factor. This cable section was offset by a distance of at least 1.5 m from the analyser to account for the length of cable outside the analyser and to avoid local effects at the chamber entry. Selected data was then extracted at points 250 mm apart, resulting in data being acquired from 13 positions along the cable section under consideration. Readings at 10 min increments over the heating and cooling cycles resulted in 19 datapoints for a given position for each 3 h long temperature step. However, the datapoints at the beginning and end of each step (the first and last 15 min) were neglected to mitigate transient effects hence 16 datapoints (from 15–165 min) were used in the analysis of the calibration factor. This was repeated for each of the 6 increasing and 6 decreasing temperature steps. The details of the spatial and time increments used are given in Table 2.

**Figure 6:**
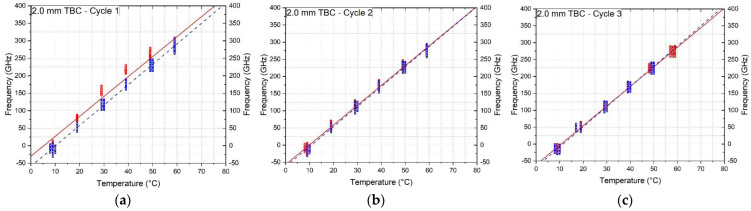
Heating and cooling calibration datapoints and linear fit of the 2.0 mm tight buffered cable with polyurethane coating, (**a**) first cycle, (**b**) second cycle and (**c**) third temperature cycle.

A linear regression was then performed on the extracted datapoints to obtain the best fit temperature calibration factor for each heating and cooling cycle. Figure 6 shows the datapoints and the slopes of the heating cycle (red solid line) and the cooling cycle (blue dashed line) for the 2.0 mm tight buffered cable for all three temperature cycles. This cable was chosen for illustrative purposes and the same methodology was applied to the other cables. In all cases, during the first temperature cycle the frequencies during cooling were lower than those measured during heating but these differences reduce by the third temperature cycle (see Figure 6a–c). This suggests that pre-heating of the cables would result in a better convergence of the heating and cooling calibration coefficients. The obtained temperature coefficients for the three heating and cooling cycles in GHz/°C are presented in Table 4 along with the standard deviations and percentage differences. The average of the increasing and decreasing temperature calibration factor for the third cycle is also indicated. The difference between heating and cooling for the third temperature cycle was lowest for the 0.9 mm tight buffered cable (TBC) with a difference of 0.09%, followed by the bare fibre with a 0.14% difference and the 3.2 mm ribbed TBC with a 0.19% difference. The largest of 3.05% was for the 2.0 mm TBC. The loose tube cables (LTC) show a difference of 0.27% for the 3.0 mm air and 0.72% for the 3.8 mm gel filled LTC during the third cycle.

### 3.3. Calibration Factor for Humidity Exposure

To evaluate the humidity calibration factor, the datapoints were obtained from the same 13 positions along the 3.0 m cable section as used for the temperature calibration procedure (see Table 2). For these 13 positions along the cable, data were extracted every 10 min during humidity exposure. However, since each humidity step lasts for 30 h, and the first and last 5 datapoints were neglected, this results in 171 datapoints per humidity level for each of the 4 increasing and 4 decreasing humidity levels.

**Figure 7:**
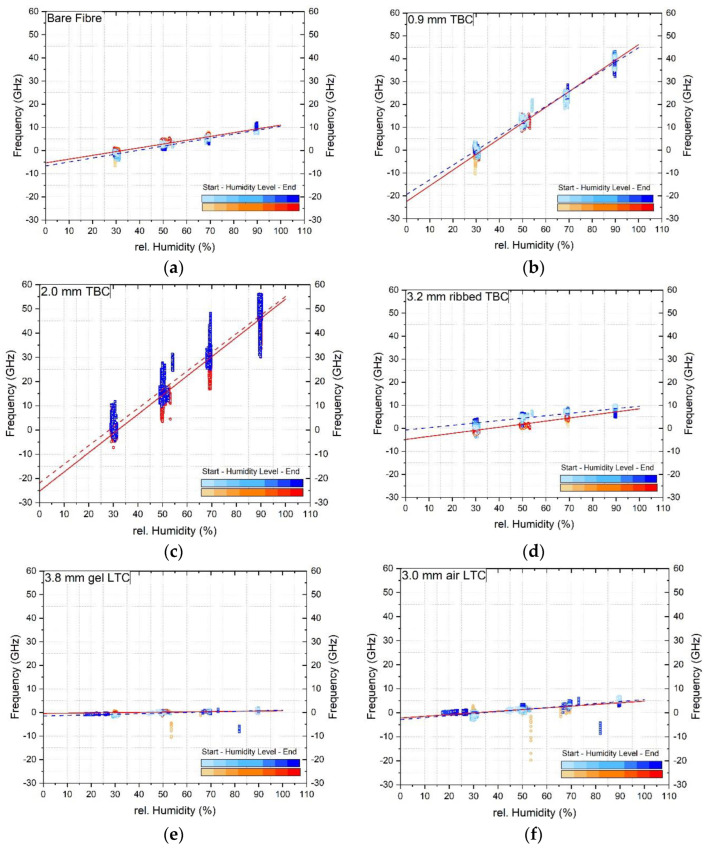
Increasing and decreasing humidity calibration datapoints and linear fit of (**a**) bare fibre, (**b**) 0.9 mm tight buffered, (**c**) 2.0 mm tight buffered, (**d**) 3.2 mm ribbed tight buffered, (**e**) 3.8 mm gel filled loose tube and (**f**) 3.0 mm air filled loose tube cable. The red line represents the increasing, the blue line the decreasing humidity level. The lighter colours refer to datapoints at the beginning (zero hours) whereas the darker colours indicate points as the end (30 h) of each humidity level.

Figure 7 shows the datapoints and slopes of the increasing and decreasing humidity cycles for all the six tested cables. The 900 μm and 2.0 mm tight buffered cables show the highest sensitivity to humidity change whereas the loose tube cables (3.8 mm gel filled and 3.0 air filled) show the lowest sensitivity. This was attributed to the presence of a stainless steel inner tube and conduit (see Table 1), which protect the fibre core from moisture ingress. The obtained increasing and decreasing calibration factors and their standard deviations are presented in Table 5. The average calibration factor over the combined increasing and decreasing cycle is also indicated.

## 4. Frequency Drifts and Fluctuations under Sustained Conditions

The slope of the linear regression line (calibration factor) represents the best fit through the selected datapoints. The deviation from the best fit is indicative of the scatter of the datapoints at each step. The scatter is smallest for the strain calibration graphs (Figure 4), somewhat greater in the temperature calibration graphs (Figure 6) and highest for the humidity calibration graphs (Figure 7. Since the datapoints reflect data taken from time and spatial intervals it is of interest to evaluate possible origins of this scatter by analysing the time stability (time-dependent drift under sustained conditions) and the length stability (differences along the cable length). This was done in the following for the temperature and humidity data since these parameters had the highest scatter and were held at constant levels for 3 and 30 h, respectively.

### 4.1. Frequency Drifts and Fluctuations

**Figure 8:**
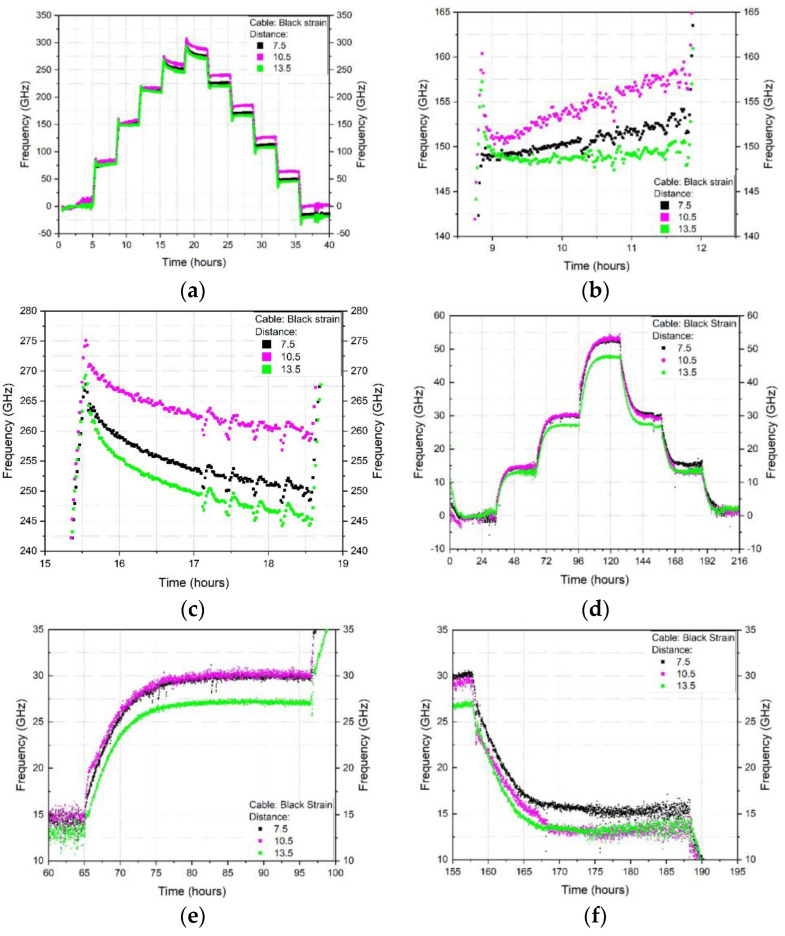
2.0 mm tight buffered cable results for (**a**) 1st heating-cooling cycle (**b**) temperature drift at 40 °C over 3 h (**c**) temperature drift at 60 °C over 3 h (**d**) increasing and decreasing humidity (**e**) humidity drift at increasing 30% rel. H over 30 h and (**f**) humidity drift at decreasing 30% rel. H. over 30 h.

In Figure 8a, the first heating and cooling cycle (10–60–10 °C) of the 2.0 mm tight buffered cable is plotted against time. The coloured lines represent the frequency shift at three different locations along the cable (at 7.5, 10.5 and 13.5 m of the 19 m long cable). This figure shows that, depending on the temperature level, the frequency either increases or decreases over time for a constant temperature. A more detailed view of the frequency drift over time for two individual temperature steps (30–40 °C and 50–60 °C) is then shown in Figure 8b,c. Furthermore, the changes in frequency during heating do not match those during cooling which would contribute to differences between the heating and cooling temperature calibration factors. A frequency drift is also observed during humidity exposure and is presented in Figure 8d–f for the 2.0 mm tight buffered cable at the same locations along the cable. Two humidity steps (30–40% and 40–50%) are shown in detail in Figure 8e,f. Although the 2.0 mm tight buffered cable was selected for illustrative purposes, frequency drifts under constant conditions were evident in all the tested cables albeit to different extents. The time-frequency plots for the six cables when subjected to temperature or humidity can be found in Appendix A. The plots exemplify that depending on the step, the frequency either increases or decreases with time.

**Figure 9:**
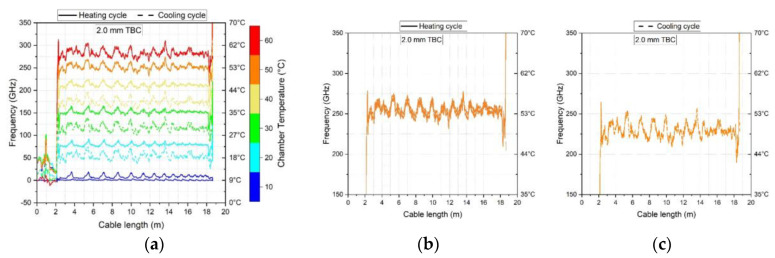
Length stability of the 2.0 TBC cable exposed to different temperature levels. (**a**) frequency shift versus cable position during heating and cooling (**b**) shifts associated with three time points at 50 °C during heating (**c**) shifts associated with three time points at 50 °C during cooling.

The response is also location dependent as there are differences between the selected locations of 7.5, 10.5 and 13.5 m e.g., as observed in Figure 8b,c,e,f. This indicates that frequency fluctuations occur along the cable. These fluctuations can be seen more clearly in Figure 9 where the frequency response along the cable length is plotted for the 2.0 mm TBC (for the full results, please see Appendix B). Hence the position along the cable where the data is extracted may also influence the readings even when the cable is exposed to the same temperature or humidity loading. This can be seen in detail in Figure 9b where the readings for three time points during the heating or cooling 50 °C temperature step are presented. The shape of the three time steps do not significantly differ from each other and show similar peaks and valleys along the cable length at similar positions. But the magnitude of the frequency between heating and cooling cycle varies. This means that depending on the analysed position along the cable one could get different readings, from a valley or a peak and in addition could record different values depending on the temperature situation (heating or cooling phase).

To investigate the aforementioned phenomena, the frequency drift over time and frequency fluctuations along the cable length were explored further. An analysis of the impact on the temperature and moisture measurement accuracy was then undertaken.

### 4.2. Frequency Drift with Time and Temperature

**Figure 10:**
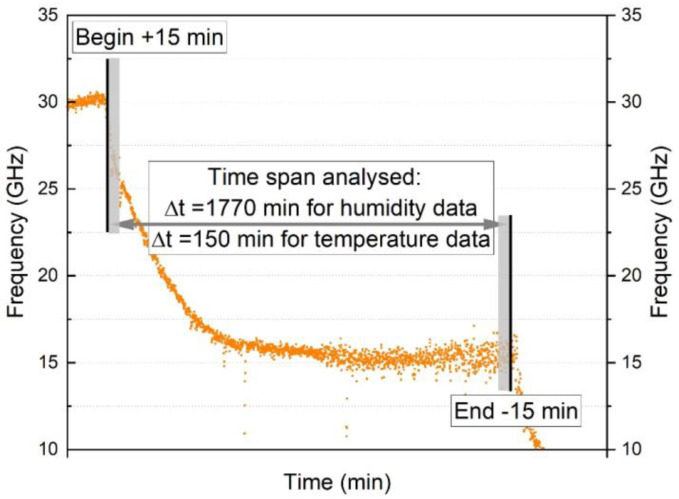
Frequency drift over time during a temperature and humidity step. For the drift analysis the first and last 15 min were neglected, hence 150 min of each temperature step and 1770 min of each humidity step were considered. The grey area represents the 10 min time span in which the mean value was taken to compare to each other.

The time- and temperature-dependent frequency drift under constant exposure conditions was assessed by selecting data using the sampling time increment of 1 min. An example of the resulting data is shown in Figure 10. Since transient frequency spikes were often observed at the beginning of each temperature step (see also Figure 8b,c), the readings from 0–15 min and 165–180 min were disregarded. The frequency readings during the period from 15–165 min (150 data points) were averaged to obtain the mean frequency for a given sustained temperature. In addition, the readings at 1 min intervals between 15–25 min and also between 155–165 min were averaged and compared to provide an indication of the magnitude of the frequency drift with time. The time spans of 10 min which were averaged are shown indicatively in Figure 10 as grey areas. To provide a better indication of the measurement parameters of interest, the average frequencies and frequency drifts were converted to temperatures using the average calibration factor shown in the 6th column in Table 4 for a given cable type. This was done for 3 different locations along the cable length, as summarised in Table 6. The length analysed was typically 6000 mm, with the exception of the bare fibre (2500 mm) and the 0.9 mm TBC cables (4000 mm) due to the shorter lengths of these cables. The same procedure, cable positions and time steps were used to analysis the time- and temperature-dependent drift for the humidity dataset. However, since each humidity step lasted for 30 h (1800 min) the average frequency was calculated over a time span between 15–1785 min (as shown indicatively in Figure 10 and Table 6) and in the conversion from frequency to relative humidity, the average moisture calibration factor in column 5 in Table 5 was used.

### 4.3. Frequency Fluctuations along the Cable Length

To probe the frequency fluctuations along the cable length, readings at 2.6 mm intervals (equivalent to the FOS gauge length) along the cable length were extracted. The cable lengths over which these were analysed are given in Table 6. The frequency fluctuations were converted to temperature or humidity using the average calibration factors for each cable type presented in Table 4 or Table 5 respectively. This allows for a better comparison of the length stability across the different cable types as the frequency shift is not as meaningful due to the varying sensitivity of the cables to temperature or humidity.

**Figure 11:**
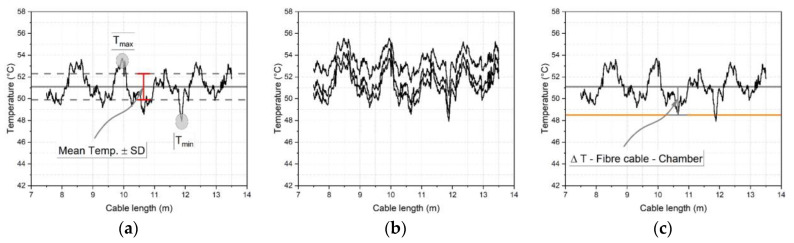
Length stability analysis, (**a**) fluctuations along the cable length, shown indicatively for the heating cycle (**b**) fluctuations for three selected times (**c**) averaging approach for a given sustained temperature.

To illustrate the subsequent analysis approach, the 2.0 mm tight buffered cable temperature results are used (see Figure 11). After applying the appropriate calibration factor to frequency data such as shown in Figure 9, a plot of the temperature fluctuation along the cable was obtained for a given time. There are various peaks at each temperature level so the minimum *T_min_* and maximum *T_max_* temperature as well as the mean temperature, *T_mean_*, were evaluated (see Figure 11a). This was calculated for three selected times during each sustained temperature (at 15 min, 90 min and 165 min) (see Figure 11b). The average *T_max_, T_min_* and *T_mean_* and the standard deviations for the three times were then used to give an indication of length fluctuation and time dependent drift effects. This analysis was repeated for each temperature level (10–60–10 °C) and for each heating and cooling cycle (cycles 1–3) such that any differences between heating and cooling could be assessed. A similar procedure was applied for the humidity dataset where the same cable positions and increments were chosen. However, since the humidity level was held for 30 h (1800 min) the times selected for analysis were chosen to be at 15, 900 and 1785 min for each humidity level.

### 4.4. Chamber Humidity and Temperature Measurement

To assess the overall accuracy, the FOS derived measurements were compared with independent readings of the studied parameters obtained from the chamber thermocouple and humidity sensors. Although the chamber temperature was fairly stable, there were nevertheless small fluctuations. Furthermore, the chamber humidity levels were less well controlled and there were variations in the humidity exposure conditions. To mitigate these effects, the reference chamber temperature and humidity levels used were those recorded at the same times as the extracted FOS data. The difference between the FOS and chamber readings were then evaluated, as shown schematically in Figure 11c.

## 5. Time- and Length Dependent Temperature and Humidity Stability

Using the approaches detailed in the previous section, the time/temperature/location-dependent and time/humidity/location-dependent variations during increasing or decreasing exposure conditions were quantified for each cable.

### 5.1. Time Dependent Temperature and Humidity Drift

**Figure 12:**
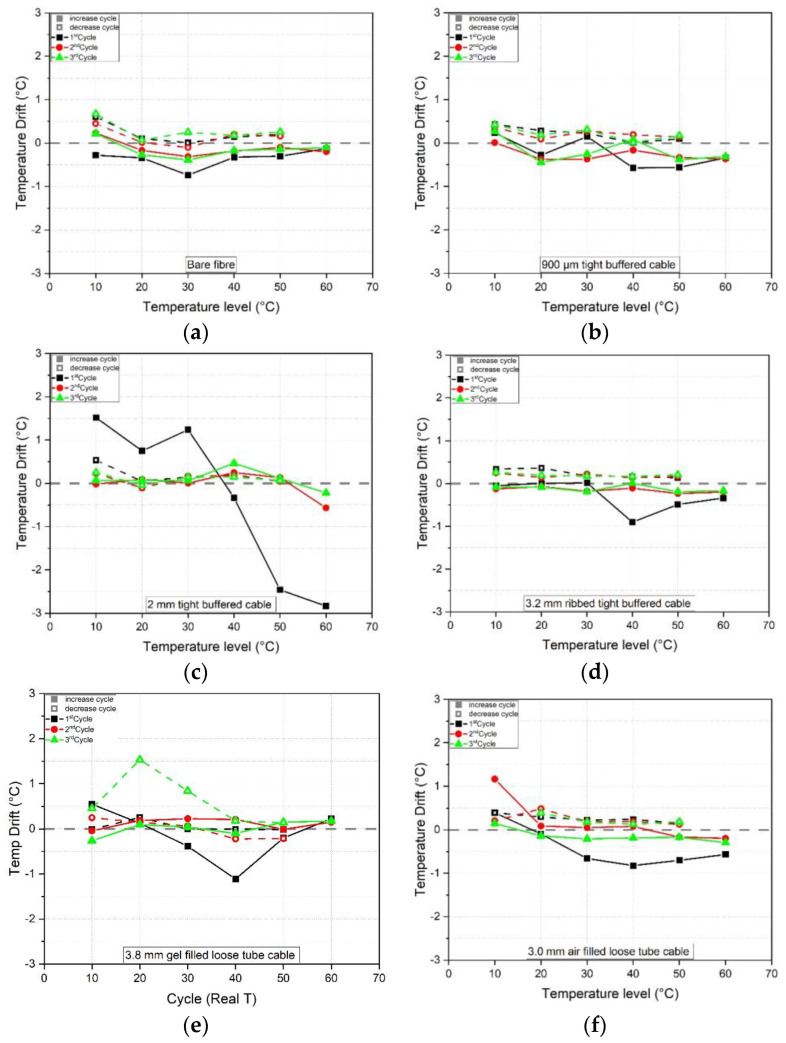
Temperature drift over time at each temperature level (10–60 °C) and cycle (1–3), (**a**) bare fibre, (**b**) 900 μm tight buffered, (**c**) 2.0 mm tight buffered, (**d**) 3.2 mm ribbed tight buffered, (**e**) 3.8 mm gel filled loose tube and (**f**) 3.0 mm air filled loose tube cable.

The results in Figure 12 show the temperature drift (by subtracting the average between 155–165 min from the average between 15–25 min for a given step) at each temperature level (10–60 °C) and for each temperature cycle (1–3) for all six cables. The black line represents the first, red line the second and green line the third cycle. The grey line represents no drift e.g., which would be associated with the same frequency reading at the beginning and end of the time period under constant conditions. Hence points above the grey line represent a downwards shift whereas points below the grey line indicate an upwards shift. In general, the temperature drift is highest during the first heating cycle (black lines) and reduces for cycles 2 and 3 (red and green lines). After the third cycle, the temperature drift is less than ± 0.5 °C except for the bare fibre (at 10 °C with a 0.6°C drift) and the 3.8 mm gel filled loose tube cable (at 20 °C with a 1.5°C drift). The drifts for each temperature step and cable type are summarised in Table 7.

**Figure 13:**
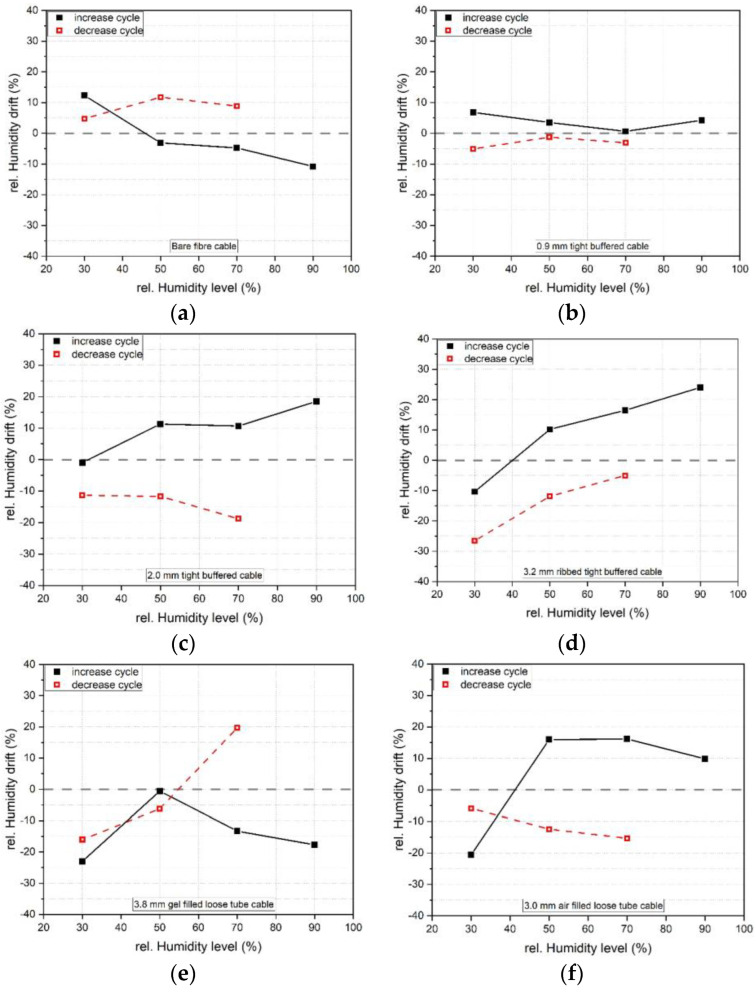
Humidity drift over time of the fibre optic cables at each humidity level (30–90% rel H), (**a**) bare fibre, (**b**) 900 μm tight buffered, (**c**) 2.0 mm tight buffered, (**d**) 3.2 mm ribbed tight buffered, (**e**) 3.8 mm gel filled loose tube and (**f**) 3.0 mm air filled loose tube cable.

The results in Figure 13 show the frequency drift over a time period (by subtracting the average between 1770–1785 min from the average between 15–25 min for a given step) at humidity levels of 30/50/70 and 90% rel. H. where the solid line represents the increasing cycle and the dashed line the decreasing cycle. Points above the zero baseline correspond to a decreasing humidity shift whereas negative values are indicative of an increase in humidity with time. All the cables show a drift over time at each humidity level. The drift depends on the cable coating and the absorption and desorption ability. In absolute terms, the 0.9 mm tight buffered cable experiences the smallest drift over time (6.8% rel. H) whereas the bare fibre with acrylate coating experiences a drift of 12.3% rel. H, the 2.0 mm tight buffered cable with polyurethane coating shows a humidity drift of 18.8% rel. H over 30 h at a 90% rel. H level. The loose tube cables (3.8 mm gel and 3.0 mm air filled cables) show high drifts and scatter at each humidity level, which is again attributed to the very low sensitivity due to the low permeability of the inner stainless steel tubes (evidenced by a low calibration factor, see Table 5). This results in a high scatter and humidity drifts for small frequency changes. A similar behaviour can be found for the 3.2 mm ribbed tight buffered cable with polyamide coating and an inner steel tube.

### 5.2. Length Dependent Temperature and Humidity Fluctuations

**Figure 14:**
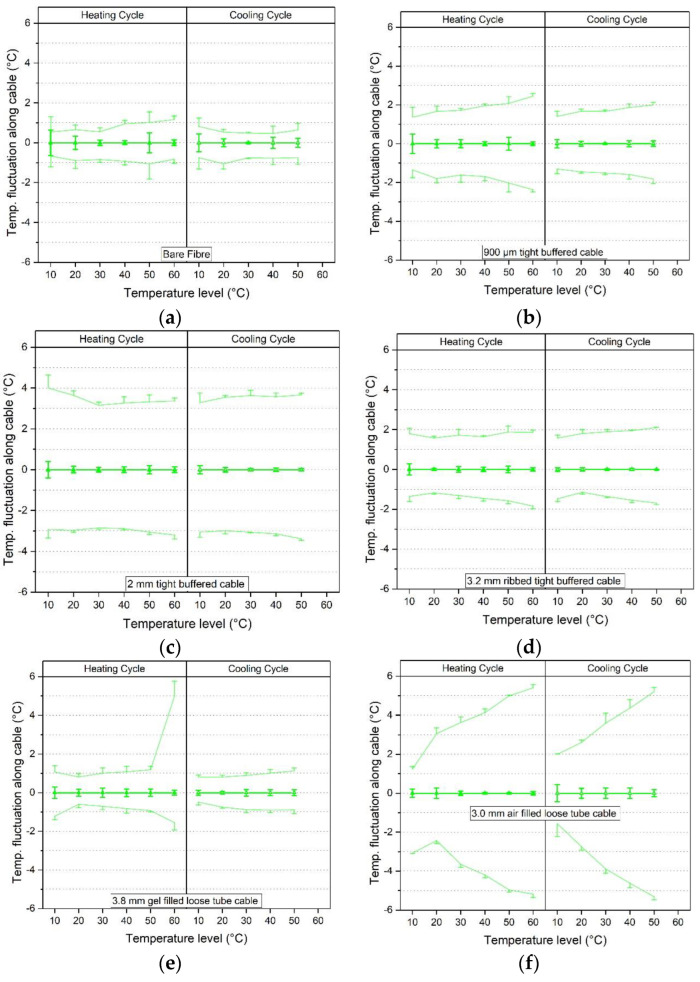
Temperature fluctuations along the fibre cable length evaluated at each temperature step for the third cycle for (**a**) bare fibre, (**b**) 900 μm tight buffered, (**c**) 2.0 mm tight buffered, (**d**) 3.2 mm ribbed tight buffered, (**e**) 3.8 mm gel filled loose tube and (**f**) 3.0 mm air filled loose tube cable.

To evaluate the stability of the readings along the cable length, the results of the temperature fluctuations along the cable length were calculated using the average and SD over the three selected time steps (15/90/165 min) and are plotted in Figure 14. The stability (fluctuation) along the cable length was calculated as described in Section 4 and Figure 11. This was done by considering each recorded measurement point along the cable length (each 2.6 mm) and taking the mean value of all those points. This mean was then the reference baseline from which the maxima and minima along the cable length was evaluated in a next step.

The comparison was made for the third heating and cooling cycle as the time-dependent drift effects were typically lowest for this cycle. Each graph represents a cable type where the left column shows the heating and the right column the cooling cycle. The results for each temperature level (10–60 °C) are shown relative to a zero line representing the mean of the three times considered, shown as thick line in Figure 14. The standard deviation of the ‘zero’ line is therefore indicative of the time-dependent drift at each temperature step. As an indication of the fluctuations along the cable, the maximum and minimum temperature along the cable length and the standard deviations of these parameters are shown as thin lines. Thus to obtain the actual measured temperature values the temperature shown on the x-axis would need to be added to the temperature fluctuation values.

The standard deviation of the average temperature along the cable length (the zero line) is within ±1 °C for all cable types. This reflects the conclusions of the previous section. However, the fluctuations (minimum and maximum peak temperatures) along the cable length vary significantly between cable types. For example, the bare fibre shows a maximum fluctuation of 1.2 °C (at 60 °C) whereas the 3.0 mm air filled loose tube cable experiences fluctuations up to 5.4 °C (the highest value being at 60 °C). The standard deviations of the minimum and maximum peak temperatures are generally fairly small suggesting that the magnitude of the peaks are not changing significantly with time under sustained conditions.

**Figure 15:**
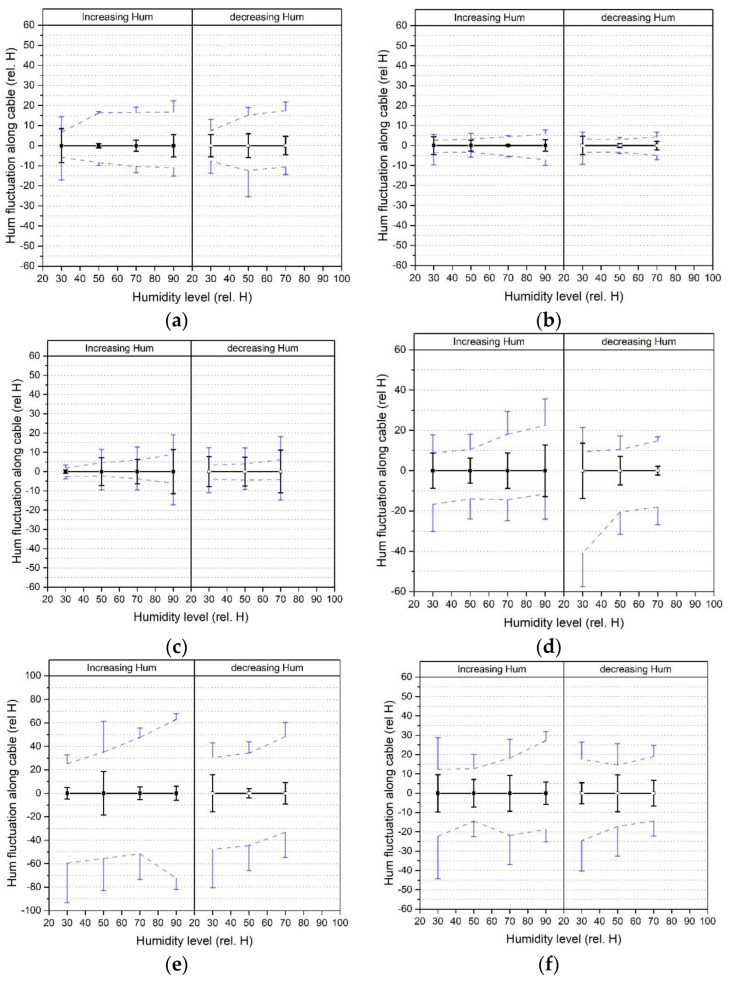
Humidity fluctuation along the fibre cable length evaluated at each humidity step for the increasing step (**left column**) and decreasing step (**right column**) for (**a**) bare fibre, (**b**) 900 μm tight buffered, (**c**) 2.0 mm tight buffered, (**d**) 3.2 mm ribbed tight buffered, (**e**) 3.8 mm gel filled loose tube and (**f**) 3.0 mm air filled loose tube cable.

In the humidity analysis, three time steps (at 15, 900 and 1785 min) were analysed. Figure 15 shows humidity fluctuations along the cable where the results for the increasing humidity (left column) are separated from the decreasing humidity cycle (right column). The results were again shown relative to a zero baseline (taken as the mean value of the frequencies at the three times considered) with the zero line mean and standard deviation, SD, shown as thick solid lines. The maximum and minimum fluctuation along the cable are shown as dashed lines. So to obtain the actual humidity magnitude, the humidity shown along the x-axis needs to be added to the mean, minimum and maximum values.

The standard deviation of the zero line demonstrates the humidity drift with time, as observed in Figure 13. The tight buffered cables show a maximum peak humidity reading in the range of between 7.1 and 41.0% rel. H. The 0.9 mm shows the lowest maximum, followed by the 2.0 mm, and finally the 3.2 mm ribbed cable. The loose tube cables show a maximum peak fluctuation of 27.2% rel. H for the air filled and 72.5% rel. H for the gel filled loose tube cable which is a result of the low sensitivity due to the steel inner tube and the decoupling of the fibre core from the coating. In Table 7, the maximum and minimum and standard deviation values are summarised. Amongst the tight buffered cables, the 0.9 mm cable shows the lowest SD with 4.6% rel. H and the 2.0 mm the highest with 11.4% rel. H. The air filled loose tube cable shows an SD of 9.6% rel. H compared to the gel filled loose tube cable with 18.6% rel. H. These fluctuations along the cable, especially the maximum peaks, are important to consider if only a short distance along the cable is analysed e.g., if only a small length is of interest and this region is close to or at the maximum peak.

### 5.3. Accuracy of FOS Measurements

**Figure 16:**
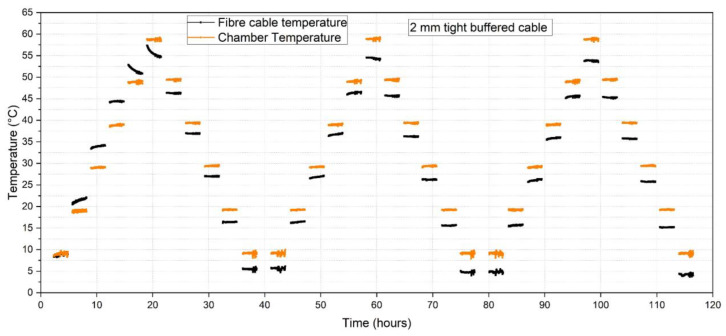
Deviation from the fibre optic cable (black line) to the chamber temperature (orange line).

**Figure 17:**
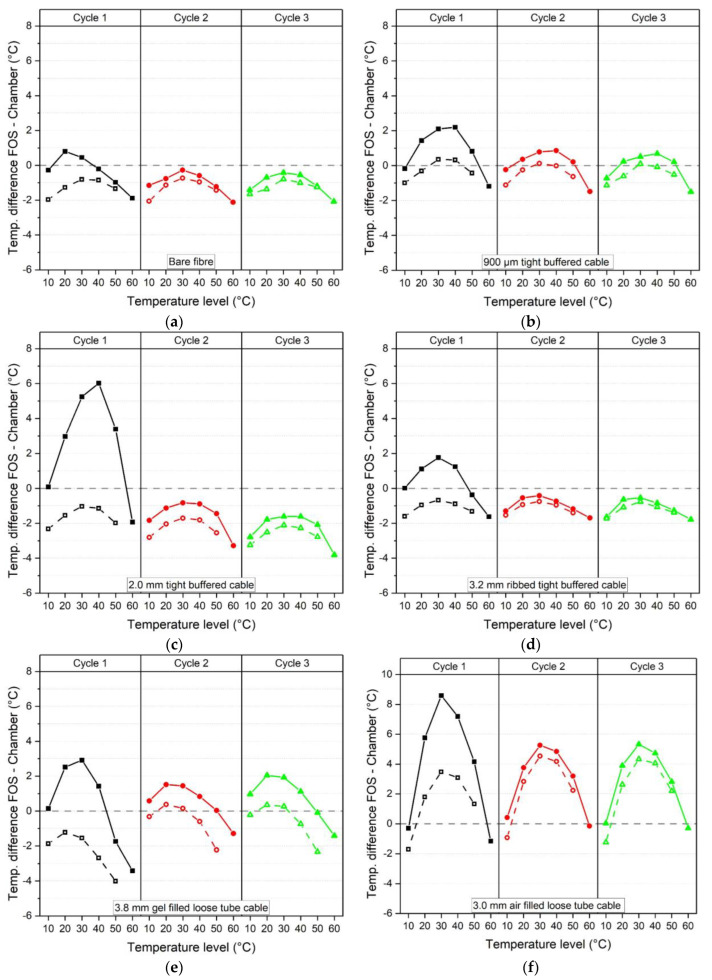
Temperature difference between fibre optic cable and chamber temperature evaluated at each temperature level. Solid lines represent increasing, dashed lines decreasing cycles, (**a**) bare fibre, (**b**) 900 μm tight buffered, (**c**) 2.0 mm tight buffered, (**d**) 3.2 mm ribbed tight buffered, (**e**) 3.8 mm gel filled loose tube and (**f**) 3.0 mm air filled loose tube cable.

**Figure 18:**
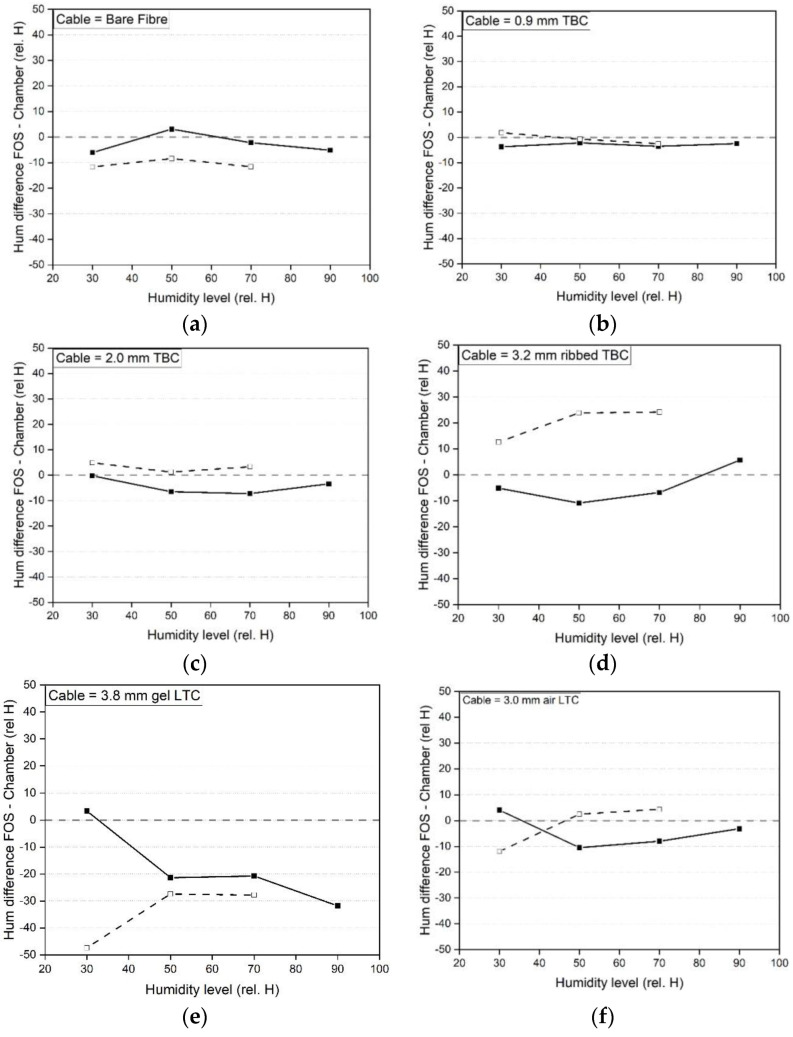
Humidity difference between fibre optic cable and chamber humidity evaluated at each humidity level, (**a**) bare fibre, (**b**) 900 μm tight buffered, (**c**) 2.0 mm tight buffered, (**d**) 3.2 mm ribbed tight buffered, (**e**) 3.8 mm gel filled loose tube and (**f**) 3.0 mm air filled loose tube cable.

The mean temperature and humidity values were compared with the chamber temperature and humidity readings as derived from the chamber thermocouples and humidity sensor respectively. Figure 16 shows illustrative temperature data for the 2.0 mm tight buffered cable for all three cycles. The orange line represents the mean chamber temperature (calculated using three time instances at the beginning, middle and end of the sustained step) and the black line is the fibre optic cable temperature back-calculated using the third cycle average heating and cooling calibration factor of 5.884 GHz/°C (see Table 4). Using this data, the difference between the FOS and chamber measurements could be calculated and plotted against the target temperature. The results for each cable type are shown in Figure 17 and Figure 18 where the solid and dashed lines represent increasing and decreasing cycles respectively. In Figure 16 it is of note that after each temperature cycle, the FOS temperature can be lower than the chamber temperature (at 10 °C). This leads to a starting point for the next cycle that lies below the grey chamber temperature line at 10 °C (solid line) at each cycle, as can be seen in Figure 17.

Figure 17 suggests that for all the cable types tested here, the first temperature cycle shows the highest deviations between the increasing and decreasing steps and also the greatest differences relative to the chamber temperature. By the third cycle the heating and cooling curves generally converge. During the third temperature cycle, the 0.9 mm tight buffered cable (TBC) shows the smallest relative difference to the chamber temperature. This cable shows a deviation of 1.5 °C at 60 °C and 1.1°C at 10 °C after cooling. These differences were also calculated as a percentage of the associated chamber temperature (see Table 8) and would result in a 2.5% and 11.3% discrepancy compared with the thermocouple readings. The highest third cycle temperature differences of 1.8 °C (at 60 °C) and 1.7 °C (at 10 °C during the cooling cycle) amongst the tight buffered cables are observed in the 2.0 mm cable. As percentages of the recorded temperature, these differ from the thermocouple readings by 3.0% and 17.2%, respectively. This demonstrates that absolute differences of a similar magnitude will introduce a more significant error when associated with lower temperatures. For the loose tube cables (LTC) the 3.8 mm gel filled cable shows fairly good agreement with the chamber temperature where differences of 3.4% (2.0 °C at 60 °C increasing level) and 4.6% (2.3 °C at the 50 °C decreasing level) during the third cycle were recorded. The air filled loose tube cable shows the highest third cycle deviations of 17.7% and 14.7% (5.3 °C and 4.3 °C) during 30 °C heating and cooling.

In Figure 18, the difference between the humidity results using the average humidity calibration factor (see Table 5)are compared with the readings from the chamber internal humidity sensor. The 0.9 mm tight buffered cable shows good agreement with the chamber humidity followed by the 2.0 mm tight buffered cable. As a percentage of the chamber measurement (see Table 8), the 0.9 mm TBC 3.7% rel. H results in a 12.3% deviation from the chamber readings whereas the 7.3% rel. H difference in the 2.0 mm TBC results in a 10.3% deviation. The cables which have a steel tube (3.2 mm tight buffered, air and gel filled loose tube cables) show higher differences due to their low sensitivity to moisture.

## 6. Discussion

For all the cable types there were differences between the heating and cooling temperature calibration factors and, with one exception, these differences were the greatest during the first temperature cycle. By the third temperature cycle the differences between the increasing and decreasing calibration factors were less than 0.2% for the 0.9 mm and 3.2 mm TBC cables and 0.8% for the LTC cables suggesting that thermal pre-conditioning would mitigate these effects. The exception was the 2.0 mm TBC which unexpectedly exhibited a higher difference in the third cycle than in the first. The time-dependent changes in the FOS measurement under sustained temperature (3 h) were also cycle dependent. The maximum absolute drift over time was between 0.2 to 2.8 °C for the tight buffered cables and 0.2 to 1.5 °C for the loose tube cables (see Table 7). During the third temperature cycle the absolute maximum temperature drift over a sustained period was less than 0.7 °C for the bare fibre, TBCs and 3.0 mm air LTC. The third cycle drift of the 3.8 mm gel LTC remained relatively high at around 1.5 °C. Overall, it appears that the differences between the FOS and traditional sensor temperature measurements and the FOS temperature drift over time could mostly be reduced by pre-treating the cables e.g., by exposing the cables to multiple heating and cooling cycles before usage. However, frequency fluctuations along the cable length at a given time and temperature were also evident. These were found to be between 1.0 to 4.1 °C for the tight buffered cables and 1.5 to 7.2 °C for the loose tube cables (see Table 7). The minimum and maximum fluctuations along the cable length were found to be broadly similar for all three temperature cycles (see Appendix B) so would not be expected to change significantly with pre-treatment. When the standard deviation, SD, of the mean value along the cable (fluctuations) was considered, the loose tube cables show a maximum SD between 0.3 to 0.7 °C whereas the tight buffered cables exhibited a maximum SD of 0.3 °C to 1.4 °C. The results suggest that the mean would potentially become less accurate if fewer cable locations are considered or, in a worst case, the measurement accuracy could be compromised by the aforementioned maximum fluctuations if only a single point along the cable was evaluated and this happened to be a peak or a trough.

The 0.9 mm and 2.0 mm tight buffered cables showed the highest sensitivity to humidity changes whereas the loose tube cables showed the lowest sensitivity. This was attributed to the different coatings of the tight buffered cables (Hytrel for the 0.9 mm and polyurethane coating for the 2.0 mm TBCs) and the presence of stainless steel tubes with a low permeability in the loose tube cables. Hence the FOS cable composition had a direct influence on the propensity for humidity measurement. In terms of the time-dependent measurement stability over a period of 30 h under sustained humidity conditions, the 0.9 mm tight buffered cable with a Hytrel coating showed a humidity drift of less than 7% rel. H whereas the humidity drift in the polyurethane coated 2.0 mm TBC was 18.8% rel. H. The bare fibre with its acrylate coating shows a drift of 12.3% rel. H. This suggests that the Hytrel coating facilitates faster adsorption and desorption and therefore results in a more stable measurement of moisture. However, further multiple adsorption and desorption cycle studies would be required to confirm these findings.

The obtained results show that there are discrepancies between the FOS-derived measurements and traditional sensors (thermocouples and moisture sensors). In absolute terms these were in the range of 1.0 and 6.0 °C or 2.5% and 10.9% rel. H for the tight buffered cables and between 1.5 to 8.7 °C or 10.5 to 47.3% rel. H for the loose tube cables (see Table 8 first cycle). For the TBCs, the FOS temperature measurement as a percentage difference from that of the chamber was generally greatest at lower temperatures (10 °C) when the temperature difference was a higher proportion of the baseline temperature. In contrast, the highest percentage differences for the LTCs were at 30 °C or 50 °C. The FOS humidity measurements deviated from those of the chamber but overall the 0.9 mm tight buffered cable showed fairly good agreement with the chamber humidity (deviations of less than 12.3%). The chamber humidity measurement itself fluctuated by 6% (see Figure 3c) and so this highlights the challenges in the accurate measurement of humidity.

## 7. Conclusions

This paper presents an in-depth analysis of time- and location- dependent stability of FOS optical backscatter reflectometer measurements under stepped increments of sustained temperature or humidity conditions. To ascertain the influence of the cable composition, a 0.125 mm bare fibre with an acrylate coating, three (0.9 mm, 2.0 mm and a 3.2 mm ribbed) tight buffered (TBC) cables and a gel (3.8 mm) or air (3.0 mm) filled loose buffered (LBC) cables were investigated. The cables were exposed to strain (0–2000–0 με), temperature (10–60–10 °C) and moisture (30–90–30% rel. H) cycles and the recorded frequency shifts were used to infer the measured strain, temperature or moisture. The data was also compared with independent readings of these parameters to evaluate the measurement accuracy.

All the tight buffered cables showed a high sensitivity to strain changes and exhibited a fairly consistent behaviour between the increasing and decreasing loading paths. The increasing and decreasing strain correlation factors of the TBC cables differed between 0.7 to 2.7%. As expected, the loose tube cables (LTC) show smaller frequency shifts due to strain. During unloading (2000–0 με) the LTC cables exhibit a plateau in the frequency below strains of 1500 με (gel filled) and 600 με (air filled) which can be considered to be indicative of a strain free window.

When subjected to temperature cycling, the best fit calibration factors differed between the heating and cooling cycles for all the cables tested. This difference reduced from the first to the third temperature cycle. Therefore, pre-heating of the fibre optic cables before usage is recommended to reduce the difference between the heating and cooling calibration coefficients.

When the fibre optic cables were exposed to a constant temperature level for 3 h a frequency drift was observed. The results show that, in general, the frequency drift was highest at high temperatures and during the first heating cycle (up to 2.8 °C for the 2.0 mm TBC). This reduced in subsequent temperature cycles.

Frequency peaks along the length of the fibre optic cables were observed at each temperature and humidity level. An analysis of the longitudinal stability was conducted by examining the maximum and minimum frequency peaks and converting these into temperatures and humidity. Frequency fluctuations along the cable length were observed and found to be between 1.0 to 4.1 °C or 5.6 to 40.9% rel. H for the tight buffered cables and 1.5 to 7.2 °C or 2.6 to 72.5% rel. H for the loose tube cables respectively

The accuracy of the FOS averaged temperature measurements was benchmarked using the recorded chamber temperatures. After the third heating-cooling cycle temperature differences of up to 17.7% (for the 3.0 mm air filled LTC) and up to 4.6% (gel filled LTC) between the FOS inferred measurements and chamber temperature were found. For the tight buffered cables the difference was up to 32.4% in the 2.0 mm TBC whereas for the 0.9 mm TBC it was 11.3%. This highlights the importance of the cable selection and pre-treatment in cases where multiple heating-cooling cycles are monitored with fibre optic cables.

For moisture exposure, the 900 μm and 2.0 mm tight buffered cables showed the highest sensitivity to humidity changes whereas the loose tube cables (the 3.0 air filled and 3.8 mm gel filled) showed the lowest sensitivity due to their construction and inner stainless steel tubes. The results suggested that the 900 μm and 2.0 tight buffered cables could be used as moisture sensors.

The results of this study demonstrate that FOS cables need to be selected carefully depending on the project specifications since the timeframe, increasing-decreasing temperature cycles and humidity exposure all influence the fibre optic cable measurement repeatability and accuracy. Consideration of these effects is of particular importance for measurements to capture indicators associated with the hardening (hydration) of concrete e.g., the heat of hydration and autogenous shrinkage or exposure to harsh environmental conditions. In such cases the moisture content and temperature change significantly within the concrete and hence the fibre optic cable concurrently experiences varying exposure conditions while being restrained to a certain degree by the concrete matrix. This interaction is complex but needs to be quantified to accurately measure temperature, humidity and strain within concrete.

## Figures and Tables

**Table 1:** Cable types, parameters and configurations.

Cable Type	Outer Dia-Meter (mm)	Coating or Sheath Material	Weight (kg/km)	Min Bend Radius (mm)	Total Cable Length (m)	Cable Length in Chamber (m)
Bare fibre	0.25	250 μm acrylate	n.a	n.a	4.50	1.1–4.5
0.9 mm TBC ^1^	0.9	Hytrel (TPC-ET thermoplastic elastomer)	0.9	20	5.25	1.0–5.25
2.0 mm TBC ^1^	2.0	Hytrel polyurethane	4.1	30	18.50	2.1–18.5
3.2 mm ribbed TBC ^1^	3.2	stainless steel tube/ structured polyamide	10.5	48	9.70	1.3–9.7
3.0 mm air filled LTC ^2^	3.0	air filled steel conduit LSZH ^3^ thermoplastic	30	30	9.70	1.1–9.7
3.8 mm gel filled LTC ^2^	3.8	gel filled stainless steel tube/polyamide	26	57	9.70	1.7–9.7

^1^ TBC—Tight buffered Cable. ^2^ LTC—Loose tube cable. ^3^ LSZH—low-smoke-zero-halogen.

**Table 2:** Calibration analyses: spatial and time increments used for the linear regression analysis.

Datapoints Used for Each Sustained Step	Analysis Cable Length (mm)	Spacing between Sampled Data Points for Analysis (mm)	# of Length Data Points	Duration*, t_total_*_,_ of Each Step (min)	Increment, Δ*t*, within Each Time Step (min)	Analysed Time from-to (min)	# of Time Data Points
Strain	900 * 1500	150 * 250	6 * 6	2 * 2	1/60 * 1/60	0–2 * 0–2	12 * 12
Temperature	3000	250	13	180	10	15–165	16
Humidity	3000	250	13	1800	10	50–1750	171

* Bare fibre.

**Table 3:** Strain calibration factor for tight-buffered and loose-tube cables.

	Strain Calibration Factor (GHz/µε)		Difference [%]	Average Strain Calibration Factor
Fibre Type	Increasing	Decreasing	Increasing-Decreasing Cycle	Increasing-Decreasing Cycle
Bare fibre	0.150 ± 9.04 × 10^−5^	0.149 ± 6.48 × 10^−5^	0.7	0.150
0.9 mm TBC	0.148 ± 5.04 × 10^−5^	0.152 ± 7.03 × 10^−5^	2.7	0.150
2.0 mm TBC	0.153 ± 5.27 × 10^−5^	0.154 ± 2.78 × 10^−5^	0.7	0.154
3.2 ribbed TBC	0.148 ± 1.10 × 10^−4^	0.146 ± 2.21 × 10^−4^	1.4	0.147
3.8 gel LTC	0.022 ± 1.22 × 10^−4^	0.016 ± 2.86 × 10^−4^	27.3	0.019
3.0 air LTC	0.079 ± 1.36 × 10^−4^	0.085 ± 6.05 × 10^−4^	7.6	0.082

**Table 4:** Temperature calibration factors for tight-buffered and loose-tube cables.

Fibre Type	Cycle	Temperature Calibration Factor (GHz/°C)		Difference Heating-Cooling Coefficient	Average Temperature Calibration Factor Increasing- Decreasing Cycle (GHz/°C)
		Heating Coefficient	Cooling Coefficient		
Bare Fibre	1	1.382 ± 0.002	1.441 ± 0.002	4.27%	
	2	1.426 ± 0.002	1.434 ± 0.002	0.56%	
	3	1.442 ± 0.002	1.444 ± 0.002	0.14%	1.443
0.9 mm TBC	1	4.509 ± 0.011	4.605 ± 0.007	2.13%	
	2	4.608 ± 0.009	4.633 ± 0.007	0.54%	
	3	4.645 ± 0.008	4.641 ± 0.007	0.09%	4.643
2.0 mm TBC	1	5.697 ±0.021	5.836 ± 0.011	2.44%	
	2	5.764 ±0.011	5.828 ± 0.011	1.11%	
	3	5.795 ±0.011	5.972 ± 0.014	3.05%	5.884
3.2 mm ribbed TBC	1	4.580 ± 0.009	4.792 ± 0.007	4.63%	
	2	4.773 ± 0.007	4.776 ± 0.007	0.06%	
	3	4.793 ± 0.007	4.802 ± 0.007	0.19%	4.798
3.8 mm gel LTC	1	1.596 ± 0.005	1.679 ± 0.005	5.20%	
	2	1.676 ± 0.003	1.674 ± 0.004	0.12%	
	3	1.674 ± 0.004	1.662 ± 0.004	0.72%	1.668
3.0 mm air LTC	1	2.520 ± 0.016	2.600 ± 0.012	3.17%	
	2	2.560 ± 0.011	2.608 ± 0.012	1.88%	
	3	2.592 ± 0.012	2.585 ± 0.012	0.27%	2.589

**Table 5:** Humidity calibration factors for tight-buffered and loose-tube cables.

	Moisture Calibration Factor (GHz/% rel. H)		Difference [%]	Average Moisture Calibration Factor (GHz/% rel. H)
Fibre type	Increasing	Decreasing	Increasing-Decreasing Cycle	Increasing-Decreasing Cycle
Bare fibre	0.163 ± 5.02 × 10^−4^	0.172 ± 3.88 × 10^−4^	5.5	0.168
0.9 mm TBC	0.687 ± 8.92 × 10^−4^	0.643 ± 9.22 × 10^−4^	6.4	0.665
2.0 mm TBC	0.792 ± 0.10 × 10^−4^	0.770 ± 0.10 × 10^−4^	2.8	0.781
3.2 ribbed TBC	0.133 ± 4.49 × 10^−4^	0.104 ± 5.53 × 10^−4^	21.8	0.119
3.8 gel LTC	0.012 ± 1.18 × 10^−4^	0.025 ± 2.01 × 10^−4^	108.3	0.019
3.0 air LTC	0.070 ± 3.91 × 10^−4^	0.083 ± 4.25 × 10^−4^	18.6	0.077

**Table 6:** Frequency drift and length stability analysis: Cable positions and time spans used for temperature and humidity.

Datapoints Used for Each Sustained Step	Analysis Length (mm)	Increment, Δ*L,* along Cable Length (mm)	# of Length Data Points	Duration*, t_total_*, of Each Step (min)	Increment, Δ*t*, within Each Time Step (min)	Analysis Times from-to (min)	# of Time Data Points
Frequency drift with time							
Temperature	2500 * 4000+ 6000	1250 2000 3000	3	180	1	15–165	150
Humidity	2500 * 4000+ 6000	1250 2000 3000	3	1800	1	15–1785	1770
Frequency fluctuation along length							
Temperature	2500 * 4000+ 6000	2.6 2.6 2.6	2308 2308 2308	180	75	15–165	3
Humidity	2500 * 4000+ 6000	2.6 2.6 2.6	2308 2308 2308	1800	875	15–1785	3

* Bare fibre. +0.9 mm TBC.

**Table 7:** Analysis of humidity and temperature including the max/min drift, max/min peaks along the length and max standard deviation, SD (shown in shaded cells).

Cable	Max/Min Humidity Drift over 30 h	Max/Min Peak Humidity along Cable	Max. SD Humidity Fluctuation along Cable	Cycle	Max/Min. Temp Drift over 2.5 h	Max/Min Peak Temperature Along Cable	Max. SD Temp Fluctuation Along Cable
	[% rel. H]	[% rel. H]	[% rel. H]		[°C]	[°C]	[°C]
Bare fibre	12.31 ± 2.94 −10.78 ± 0.79	17.47 ± 4.27 −12.41 ± 13.05	±8.45	1	0.60 ± 0.16 −0.73 ± 0.23	1.18 ± 0.55 −1.06 ± 0.38	±0.78
				2	0.45 ± 0.09 −0.31 ± 0.13	1.01 ± 0.18 −0.98 ± 0.19	±0.66
				3	0.67 ± 0.14 −0.39 ± 0.10	1.16 ± 0.18 −1.06 ± 0.27	±0.64
0.9 mm TBC	6.80 ± 0.89 −5.08 ± 0.84	5.58 ± 2.13 −7.12 ± 2.77	±4.57	1	0.43 ± 0.04 −0.57 ± 0.23	2.34 ± 0.46 −2.43 ± 0.42	±0.80
				2	0.36 ± 0.20 −0.37 ± 0.04	2.37 ± 0.29 −2.37 ± 0.28	±0.57
				3	0.42 ± 0.06 −0.44 ± 0.07	2.45 ± 0.13 −2.37 ± 0.12	±0.49
2.0 mm TBC	18.48 ± 1.82 −18.79 ± 0.45	8.94 ± 10.19 −6.11 ± 11.09	±11.41	1	1.52 ± 1.02 −2.83 ± 0.12	3.51 ± 0.24 −3.28 ± 1.22	±1.42
				2	0.25 ± 0.20 −0.57 ± 0.05	4.07 ± 0.52 −3.32 ± 0.17	±0.34
				3	0.46 ± 0.07 −0.22 ± 0.08	4.01 ± 0.63 −3.38 ± 0.08	±0.40
3.2 mm ribbed TBC	24.03 ± 0.73 −26.53 ± 0.71	22.43 ± 13.21 −40.97 ± 16.52	±13.71	1	0.36 ± 0.09 −0.90 ± 0.13	2.02 ± 0.04 −1.80 ± 0.10	±0.32
				2	0.25 ± 0.14 −0.23 ± 0.11	2.11 ± 0.02 −1.78 ± 0.12	±0.27
				3	0.26 ± 0.13 −0.19 ± 0.01	2.10 ± 0.02 −1.83 ± 0.14	±0.28
3.8 mm gel LTC	19.69 ± 5.38 −23.00 ± 15.87	62.96 ± 4.88 −72.45 ± 9.56	±18.58	1	0.55 ± 0.15 −1.11 ± 0.10	7.23 ± 0.79 −1.74 ± 0.64	±0.49
				2	0.25 ± 0.09 −0.22 ± 0.37	5.39 ± 1.00 −1.44 ± 0.36	±0.51
				3	1.52 ± 0.10 −0.26 ± 0.11	4.97 ± 0.78 −1.54 ± 0.38	±0.29
3.0 mm air LTC	16.15 ± 3.58 −20.58 ± 3.40	27.16 ± 4.76 −24.58 ± 15.78	±9.62	1	0.39 ± 0.40 −0.82 ± 0.07	5.91 ± 0.22 −5.33 ± 0.14	±0.46
				2	1.16 ± 1.41 −0.19 ± 0.16	5.58 ± 0.42 −5.33 ± 0.14	±0.67
				3	0.39 ± 0.07 −0.29 ± 0.12	5.41 ± 0.17 −5.31 ± 0.16	±0.44

**Table 8:** Analysis of humidity and temperature deviation from the chamber reading.

Cable	Cycle Path	Max/Min. Deviation from Chamber Humidity	Humidity Level for Min/Max Deviation	Deviation from Chamber in%	Cycle	Cycle Stage	Max/Min. Deviation from Chamber Temp	Temperature Level for Min/Max Deviation	Deviation from Chamber in%
		[% rel. H]	[% rel. H]	[%]			[°C]	[°C]	[%]
Bare fibre	Increasing decreasing	−6.05 −11.0	30 30	20.1 36.7	1	Increasing decreasing	−1.88 −1.96	60 10	3.1 19.6
					2	Increasing decreasing	−2.11 −2.06	60 10	3.5 20.6
					3	Increasing decreasing	−2.07 −1.65	60 10	3.5 16.5
0.9 mm tight buffered	Increasing decreasing	−3.70 −2.49	30 70	12.3 3.6	1	Increasing decreasing	2.20 −1.00	40 10	5.5 1.0
					2	Increasing decreasing	−1.49 −1.11	60 10	2.5 11.1
					3	Increasing decreasing	−1.50 −1.13	60 10	2.5 11.3
2.0 mm tight buffered	Increasing decreasing	−7.24 4.93	70 30	−10.3 16.4	1	Increasing decreasing	6.02 −2.32	40 10	15.1 23.2
					2	Increasing decreasing	3.28 2.81	60 10	5.5 28.1
					3	Increasing decreasing	3.81 −3.24	60 10	6.4 −32.4
3.2 mm ribbed tight buffered	Increasing decreasing	−10.86 24.2	50 70	−21.7 34.6	1	Increasing decreasing	1.76 −1.60	30 10	5.9 16.0
					2	Increasing decreasing	−1.69 −1.53	60 10	2.8 15.3
					3	Increasing decreasing	−1.78 −1.72	60 10	3.0 17.2
3.8 mm gel filled loose tube	Increasing decreasing	−31.81 −47.35	90 30	−35.3 −157.8	1	Increasing decreasing	3.4 −4.0	60 50	5.7 8.0
					2	Increasing decreasing	1.51 −2.22	30 50	5.0 4.4
					3	Increasing decreasing	2.05 −2.32	60 50	3.4 4.6
3.0 mm air filled loose tube	Increasing decreasing	−10.47 −11.97	50 30	−20.9 −39.9	1	Increasing decreasing	8.68 3.48	30 30	28.9 11.6
					2	Increasing decreasing	5.26 4.53	30 30	17.5 15.1
					3	Increasing decreasing	5.32 4.33	30 30	17.7 14.4

**Figure A1:**
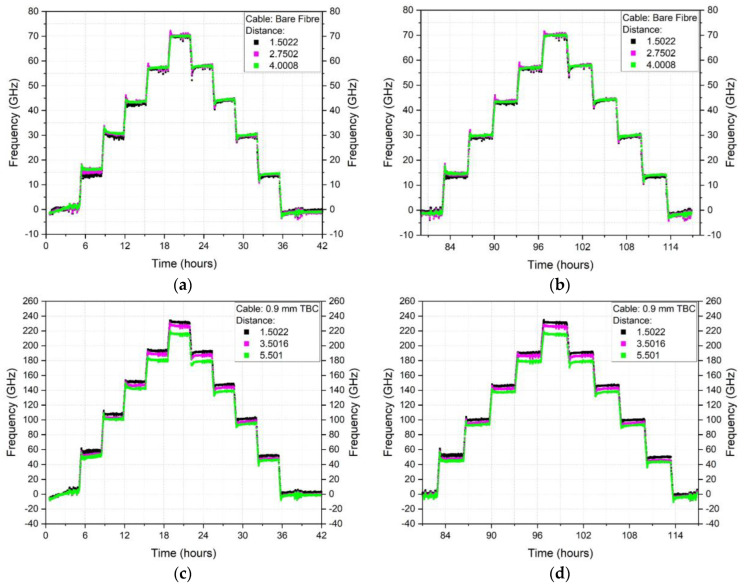

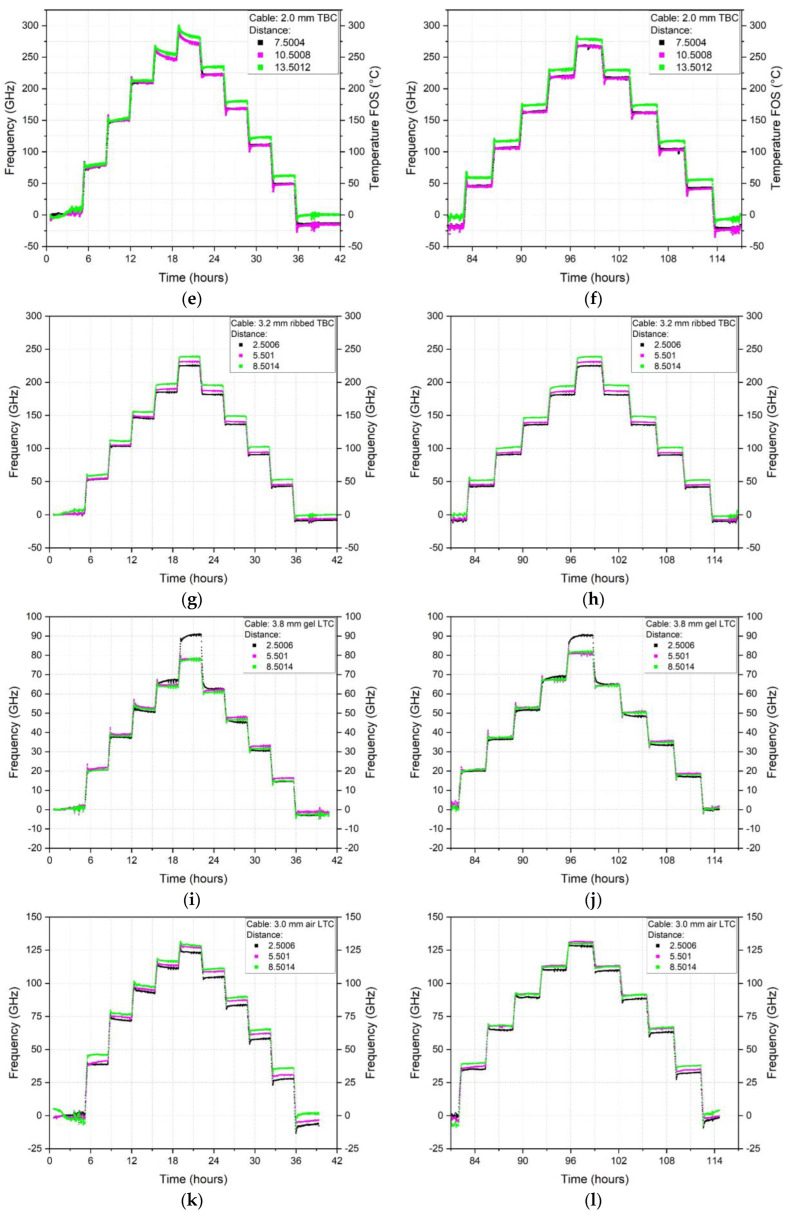
Time-Frequency plots for sustained temperature steps 10–60–10–10 °C for **bare fibre cable** (**a**) 1st heating-cooling cycle, (**b**) 3rd heating-cooling cycle; **900 μm tight buffered cable** (**c**) 1st heating-cooling cycle, (**d**) 3rd heating-cooling cycle; **2.0 mm black tight buffered cable** (**e**) 1st heating-cooling cycle, (**f**) 3rd heating-cooling cycle; **3.2 mm blue ribbed tight buffered cable** (**g**) 1st heating-cooling cycle, (**h**) 3rd heating-cooling cycle; **3.8 mm-gel filled loose tube cable** (**i**) 1st heating-cooling cycle, (**j**) 3rd heating-cooling cycle; **3.0 mm-air filled loose tube cable** (**k**) 1st heating-cooling cycle, (**l**) 3rd heating-cooling cycle.

**Figure A2:**
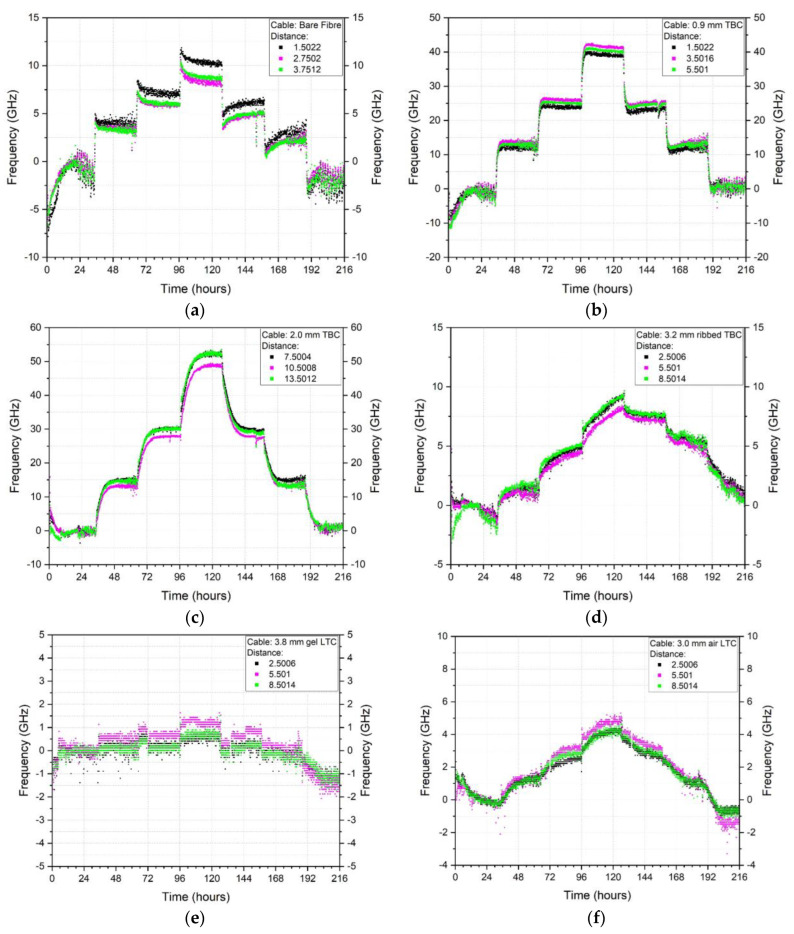
Time-Frequency plots for the time stability analysis during humidity steps of 30–90–30–20% rel. H. (**a**) **bare fibre cable** (**b**) 900 μm tight buffered cable (**c**) 2.0 mm black tight buffered cable (**d**) 3.2 mm blue ribbed tight buffered cable (**e**) 3.8 mm gel filled loose tube cable and (**f**) 3.0 mm air filled loose tube cable.

**Figure A3:**
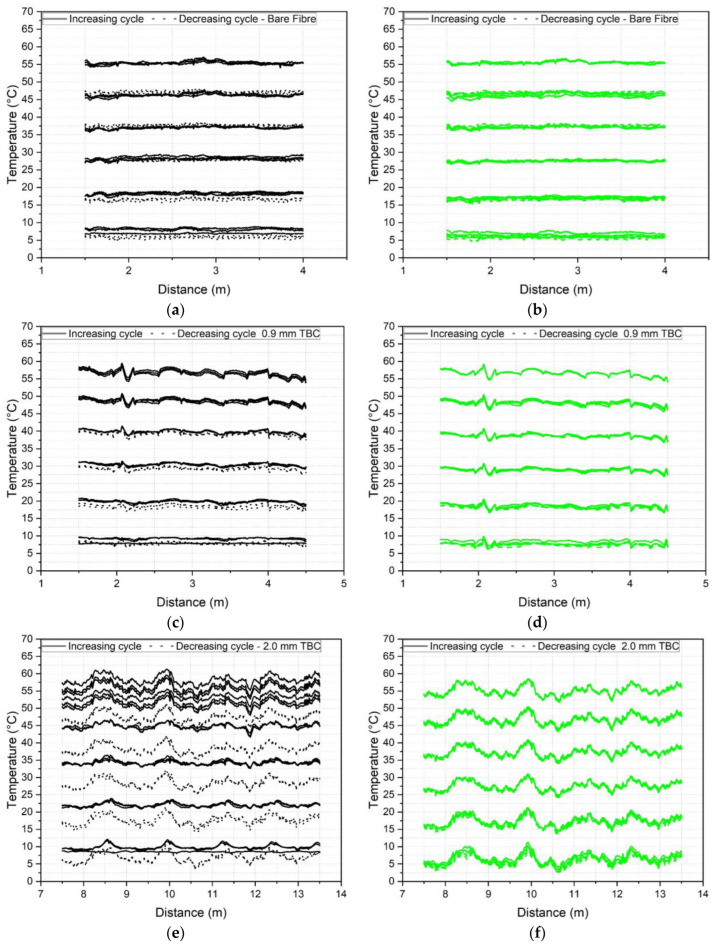

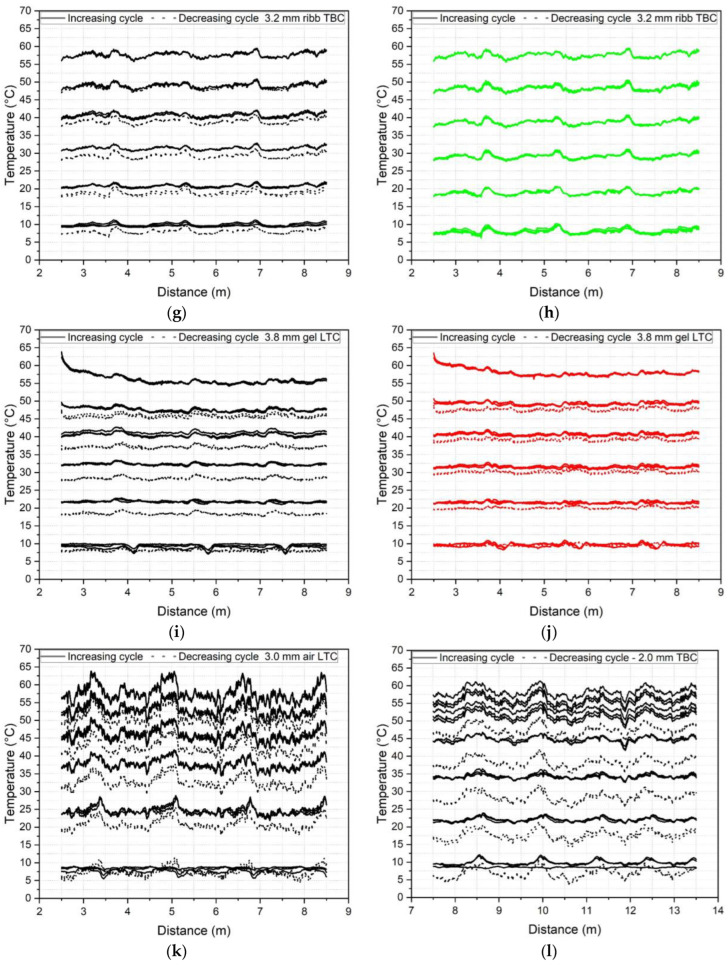
Length stability along analysed cable section for temperature steps of 10–60–10 °C for the bare fibre cable (**a**) 1st heating-cooling cycle, (**b**) 3rd heating-cooling cycle; 900 μm tight buffered cable (**c**) 1st heating-cooling cycle, (**d**) 3rd heating-cooling cycle; 2.0 mm black tight buffered cable (**e**) 1st heating-cooling cycle, (**f**) 3rd heating-cooling cycle; 3.2 mm blue ribbed tight buffered cable (**g**) 1st heating-cooling cycle, (**h**) 3rd heating-cooling cycle; **3**.8 mm-gel filled loose tube cable (**i**) 1st heating-cooling cycle, (**j**) 3rd heating-cooling cycle; 3.0 mm-air filled loose tube cable (**k**) 1st heating-cooling cycle, (**l**) 3rd heating-cooling cycle.

**Figure A4:**
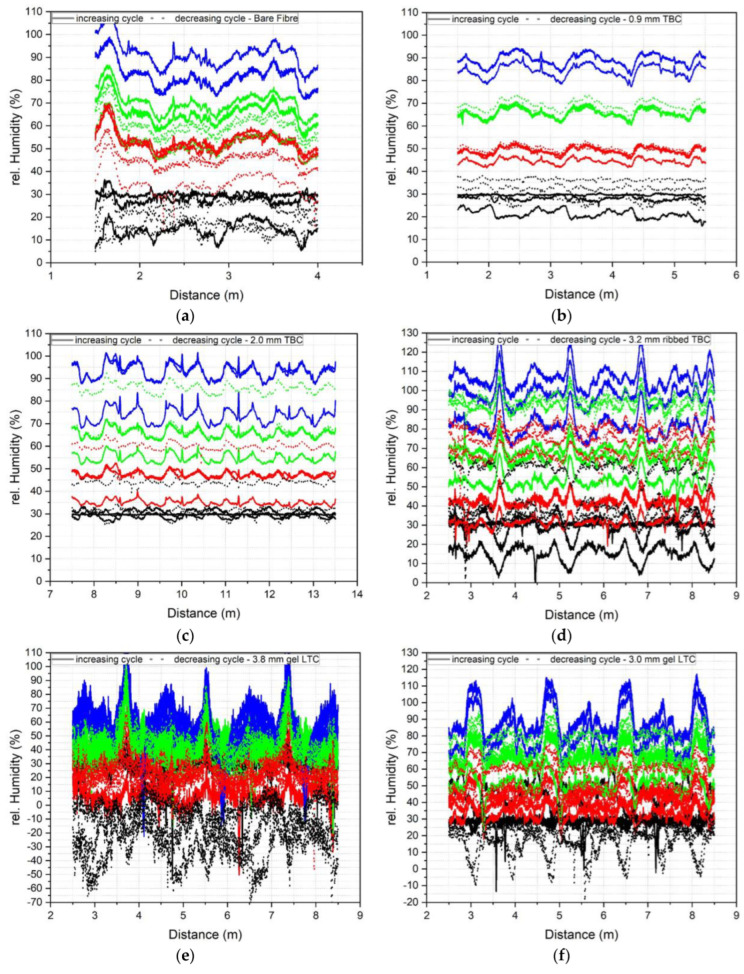
Humidity, spatial stability of (**a**) bare fibre cable (**b**) 900 μm tight buffered cable (**c**) 2.0 mm black tight buffered cable (**d**) 3.2 mm blue ribbed tight buffered cable (**e**) 3.8 mm^−2^ core loose tube (gel filled) cable (**f**) 3.0 mm^−1^ core black loose tube (air filled) cable.

## Data Availability

Not applicable.

## Appendix A. DFOS Temperature or Humidity Exposure Frequency Versus Time Plots

Temperature cycles 10/20/30/40/50/60/50/40/30/20/10 °C.

Humidity cycles 30/50/70/90/70/50/30% rel. H

## Appendix B. Longitudinal DFOS Temperature and Humidity Frequency Variations

Temperature cycles 10/20/30/40/50/60/50/40/30/20/10 °C

Humidity cycles 30/50/70/90/70/50/30% rel. H.
